# Down‐regulated HDAC3 elevates microRNA‐495‐3p to restrain epithelial‐mesenchymal transition and oncogenicity of melanoma cells via reducing TRAF5

**DOI:** 10.1111/jcmm.15885

**Published:** 2020-10-13

**Authors:** Yanbo Ma, Jincheng Duan, Xiuyan Hao

**Affiliations:** ^1^ Oral and maxillofacial surgery Linyi People's Hospital Linyi China; ^2^ Department of stomatology Linyi People's Hospital Linyi China

**Keywords:** epithelial‐mesenchymal transformation, histone deacetylase 3, melanoma, microrna‐495‐3p, oncogenicity, tumour necrosis factor receptor‐associated factor 5

## Abstract

MicroRNAs (miRNAs) are emerging biomarkers in biological processes and the role of miR‐495‐3p has been identified in melanoma, while the detailed molecular mechanisms remain to be further explored. We aim to explore the effect of histone deacetylase 3 (HDAC3) and miR‐495‐3p on epithelial‐mesenchymal transition (EMT) and oncogenicity of melanoma cells by regulating tumour necrosis factor receptor‐associated factor 5 (TRAF5). Levels of HDAC3, miR‐495‐3p and TRAF5 in melanoma tissues and pigmented nevus tissues were determined, and the predictive roles of HDAC3 and miR‐495‐3p in prognosis of melanoma patients were measured. The melanoma cells were screened and transfected with relative oligonucleotides and plasmids, and the expression of HDAC3, miR‐495‐3p and TRAF5, and phenotypes of melanoma cells were gauged by a series of assays. The relations between HDAC3 and miR‐495‐3p, and between miR‐495‐3p and TRAF5 were confirmed. HDAC3 and TRAF5 were increased while miR‐495‐3p was decreased in melanoma cells and tissues, and the low expression of miR‐495‐3p as well as high expression of HDAC3 indicated a poor prognosis of melanoma patients. Inhibited HDAC3 elevated miR‐495‐3p to suppress EMT and oncogenicity of melanoma cells by reducing TRAF5. HDAC3 particularly bound to miR‐495‐3p and TRAF5 was the target gene of miR‐495‐3p. Our results revealed that down‐regulated HDAC3 elevates miR‐495‐3p to suppress malignant phenotypes of melanoma cells by inhibiting TRAF5, thereby repressing EMT progression of melanoma cells. This study may provide novel targets for melanoma treatment.

## 1 INTRODUCTION

Skin cancer is the commonest form of cancer, and the number of diagnosed cases is increasing every year. Skin cancers are separated into non‐melanoma skin cancers and melanoma.^1^ Melanoma is the most aggressive skin cancer and accounts for >80% of skin cancer‐related deaths.^2^ Though the incidences of most cancers are reducing, the prevalence of melanoma has been steadily increased all over the world.^3^ Melanocytes are naturally occurring pigmented cells in the epidermis and malignant transformation of melanocytes induces melanoma. Melanocytes are responsible for the production of an endogenous pigment melanin that protects skin against harmful ultraviolet radiation.^4^ It is reported that exposure to the highly energetic spectrum of ultraviolet radiation is the main cause of melanoma.^5^ Although treatments for melanoma such as targeted and immune therapies have been developed recently, additional effective therapeutic strategies for melanoma are still needed.^2^

Histone deacetylases (HDACs) knock out acetyl groups, typically promoting a closed chromatin structure that constrains gene expression.^6^ HDACs are separated into four groups based on their structure and function, and HDAC3 belongs to class I.^7^ It was previously suggested that the proliferation of melanoma cells was inhibited by reduction of HDAC3,^8^ and a recent study revealed that the inhibited HDAC3 contributed to killing melanoma cells.^9^ MicroRNAs (miRNAs) are non‐coding short RNAs that regulate gene expression and negatively impact the stability and translation of mRNAs by binding to complementary sequences at 3′ untranslated region (3′UTR).^10^ It was reported that miR‐137 acted as a tumour repressor in malignant melanoma,^11^ and miR‐337 was demonstrated to be a vital negative regulator in melanoma.^12^ As one of the miRNAs, miR‐495‐3p was involved in the development of melanoma. For instance, it was identified that the elevation of miR‐495‐3p was capable of inhibiting malignant behaviours of melanoma cells,^13^ and miR‐495 was verified as a tumour repressor in melanoma.^14^ Nevertheless, relation between HDAC3 and miR‐495‐3p remains unknown. Tumour necrosis factor receptor‐associated factor family members (TRAFs) are intracellular adaptors that regulate cellular effects via binding to their cognate cellular receptors. One of the family members, TRAF5, plays an essential role in cell biological processes.^15^ It was revealed that suppression of TRAF5 was able to inhibit proliferation while induce apoptosis of melanoma cells,^16^ while the target relation between miR‐495‐3p and TRAF5 is little known.

We aim to explore the impact of HDAC3/miR‐495‐3p/TRAF5 axis on melanoma, and we supposed that HDAC3 may mediate miR‐495‐3p to regulate the biological processes of melanoma cells by modulating TRAF5.

## 2 MATERIALS AND METHODS

### 2.1 Ethics statement

Written informed consents were acquired from all patients before this study. The protocol of this study was confirmed by the Ethic Committee of Linyi People's Hospital. Animal experiments were strictly in accordance with the Guide to the Management and Use of Laboratory Animals issued by the National Institutes of Health. The protocol of animal experiments was approved by the Institutional Animal Care and Use Committee of Linyi People's Hospital.

### 2.2 Study subjects

One hundred and fifteen melanoma tissues from patients that had accepted resection in Linyi People's Hospital were collected and confirmed by histopathology. The clinical data of patients, including onset age, gender, tumour classification, tumour, node and metastasis (TNM) stage, lymph node metastasis (LNM) and distant metastasis were collected. Forty control pigmented nevi tissues that obtained from skin biopsy in Linyi People's Hospital were taken as the control tissues.

### 2.3 Cell culture

Normal melanocytes HEMa‐LP and melanoma cells A357, A875, MUM‐2B, MUM‐2C and SK‐MEL‐28 were acquired from American Type Culture Collection. HEMa‐LP, A875, A375 and SK‐MEL‐28 cells were cultured with Dulbecco's modified Eagle medium (DMEM) (Gibco Company), and MUM‐2B and MUM‐2C cells were cultured with Roswell Park Memorial Institute (RIMP)‐1640 medium (Gibco Company). Media of the 6 cell lines all contained 10% foetal bovine serum (FBS) and 1% streptomycin‐penicillin, and the cells were cultured at 37°C with 5% CO_2_. Expression levels of miR‐495‐3p, HDAC3 and TRAF5 in cells were determined using reverse transcription quantitative polymerase chain reaction (RT‐qPCR) and Western blot analysis. Two cell lines that had the lowest expression of miR‐495‐3p were selected for subsequent experiments.

### 2.4 Cell grouping

Cells were screened and classified into seven groups and, respectively, treated with HDAC3 small interfering RNA‐negative control (si‐NC), si‐HDAC3 (HDAC3 siRNA), mimics NC, miR‐495‐3p mimics, si‐HDAC3 + miR‐495‐3p inhibitors, si‐TRAF5 (TRAF5 siRNA) or TRAF5 siRNA NC (Control siRNA). The transfection was in line with instruction of Lipofectamine 2000 (Invitrogen Inc). HDAC3 siRNA, HDAC3 siRNA NC, miR‐495 mimics, miR‐495 mimics NC, TRAF5 siRNA, TRAF5 siRNA NC and miR‐495‐3p inhibitors were all synthetized by GenePharma Co., Ltd.

### 2.5 3‐(4,5‐dimethyl‐2‐thiazolyl)‐2,5‐diphenyl‐2‐H‐tetrazolium bromide (MTT) assay

Cells after transfection were collected, and the concentration was adjusted to 1 × 10^4^ cells/mL. The cell suspension was seeded and incubated for 24 hours, and each well was supplemented with 20 μL MTT solution (5 mg/mL, Sigma‐Aldrich Chemical Company) at 24 hours, 48 hours and 72 hours of the incubation, respectively, then continuously incubated for 4 hours. Cells were centrifuged with the supernatant discarded, and each well was appended with 150 μL dimethyl sulfoxide. The blue crystal was completely dissolved after shaken for 10 minutes, and then, the optical density (OD) value at 570 nm of each well was analysed by a microplate reader (FUJIFILM Wako Pure Chemical Corporation). This experiment was repeated for three times, and the results were recorded.

### 2.6 Colony formation assay

Cells were detached using 0.05% trypsin and seeded into 6‐cm culture discs at 1000 cells/well, then cultured for 12‐15 d. With the medium removed, the cells were fixed with 10 mL 4% paraformaldehyde (Sigma) for 20 minutes and stained with crystal violet dye solution (Sigma) for 30 minutes. The dried cells were photographed under a microscope, and the colonies were counted.

### 2.7 Flow cytometry

Cell cycle determination: cell concentration was modulated to 1 × 10^6^ cells/mL and the cells were centrifuged at 4°C and 2000 rpm for 10 minutes, then suspended by 1 mL phosphate‐buffered saline. Next, the cells were stained with 10 μL propidium iodide (PI) and 5 μL RnaseA (Thermo Fisher Scientific Inc) without light exposure for 30 minutes. The cell cycle distribution was gauged by a flow cytometer (BD Biosciences).

Cell apoptosis detection: cell concentration was modulated to 1 × 10^6^ cells/mL and the cells were centrifuged at 4°C and 2000 rpm for 10 minutes and then suspended by 300 μL 1 × binding buffer; thereby, the concentration was adjusted to 1 × 10^6^ cells/mL. Each well was appended with 5 μL Annexin V‐fluorescein isothiocyanate (Thermo Fisher Scientific Inc) for 15‐minutes incubation without light exposure at 2‐8°C. Cells were filtered by a 400‐mesh net, incubated with 10 μL PI at 4°C in dark for 15 minutes and added with 200 μL 1 × binding buffer, and then, the samples were put into the flow cytometer in 1 hour. The apoptosis rate of each group was calculated.

### 2.8 Hoechst 33342 staining

Detached cells were seeded into 24‐well plates at 2 × 10^4^ cells/well until the confluence reached 70%, and then, the medium was replaced by a serum‐free one for 12‐hours synchronized culture. With the medium removed, cells in each well were fixed with 1 mL 4% paraformaldehyde at 4°C for 20 minutes and stained by 1 mL Hoechst 33342 dye solution (Beyotime Institute of Biotechnology) at 37°C for 20 minutes. Apoptosis was observed under an inverted fluorescence microscope.

### 2.9 Scratch test

Cells were seeded onto 24‐well plates and cultured for 24 hours under standard conditions, then a straight scratch was made in the middle of the cell monolayer with a sterile pipette tip, and wound‐healing status was finally observed under microscopy 24 hours later. The ImageJ 1.35 software was used to analyse the distance of scratches. The wound healing was quantified as the ratio of remaining area without cell and primary wound area.

### 2.10 Transwell assay

Matrigel (Invitrogen Inc) was fused and put on ice for 1 hour. Apical chambers of the Transwell were coated with 30 μL Matrigel, and the cells were incubated for 2 hours. Cells in logarithmic growth phase were detached and resuspended, and the concentration was adjusted (dilution and stop buffer didn't contain serum). Complete medium containing 20% FBS was supplemented into 24‐well plates, and the apical chambers were appended with 200 μL cell suspension for 36‐hours incubation. The chambers were taken out and unmigrated cells were removed and fixed with −20°C methanol on ice for 10 minutes and stained with 2% crystal violet dye solution for 1 hour. Five fields of view of each chamber were selected to count the cells.

### 2.11 Western blot analysis

Cells or tissues were lysed by 1 × radio‐immunoprecipitation assay lysis buffer (Beyotime) on ice for 30 minutes and boiled at 100°C for protein denaturation. The protein fluid of cells or tissues was stored at −80°C. Prepared 10% separation gel was poured into the gel caster and added with water saturated n‐butanol, then the gel surface was flattened and the gel was placed for 30 minutes. Afterwards, the water saturated n‐butanol was removed and the upper gel was supplemented with ionized water and placed for 30 minutes. The gel device was taken out, and the gel was added with 1 × electrophoretic buffer. Each well was added with 30 μL protein fluid and protein maker. After the polyacrylamide gel electrophoresis, proteins were transferred onto membranes, which were blocked with 5% skim milk powder for 1 hour and incubated with primary antibodies HDAC3 (1:1000 and from Abcam Inc, Cambridge), TRAF5 (1:500), glyceraldehyde phosphate dehydrogenase (GAPDH, 1:10 000, both from Proteintech Group Inc), N‐cadherin and E‐cadherin (both 1:1000 and from Cell Signaling Technologies) for 1 hour and stayed overnight at 4°C. Subsequently, the membranes were incubated with relative secondary antibody for 2 hours and added with horseradish peroxidase enhanced chemiluminescent reagent, then scanned by C‐DiGit instrument and analysed by Image Studio software. GAPDH was the internal reference protein, and the ratio of grey value was used for the quantitative determination of protein analysis.

### 2.12 RT‐qPCR

Total RNA in cells or tissues was extracted by TRIzol kits (Tiangen Biotech Co., Ltd.). The RNA concentration was measured using an ultraviolet spectrophotometer and RNA was reversely transcribed into cDNA with Quant cDNA first strand synthesis kits (Tiangen Biotech). The PCR was conducted by 2 × Taq PCR MasterMix kits (Tiangen Biotech) and ABI 7900 PCR amplifier (ABI Company) was used for the measurement. The primers (Table 1) were synthetized by Shanghai Sangon Biotechnology Co., Ltd. U6 was used as the loading control of miR‐495‐3p, and GAPDH was used as the loading control of HDAC3 and TRAF5. The data were analysed using 2^‐ΔΔCt^.

**TABLE 1:** Primer sequence

Gene	Sequence
miR‐495‐3p	F: 5′‐ACACTCCAGCTGGGAAACAAACATGGTGCA‐3′
	R: 5′‐TGGTGTCGTGGAGTCG‐3′
TRAF5	F: 5′‐AACCTGACCCCAATAGCAGC‐3′
	R: 5′‐ TCAGTTAAGTCCACGGCCAC‐3′
HDAC3	F: 5′‐CACCCTATGAAGCCCCATCG‐3′
	R: 5′‐GAGACCGTAATGCAGGACCAG‐3′
U6	F: 5′‐CTCGCTTCGGCAGCACA‐3′
	R: 5′‐AACGCTTCACGAATTTGCGT‐3′
GAPDH	F: 5′‐ATGGGGAAGGTGAAGGTCG‐3′
	R: 5′‐GGGGTCATTGATGGCAACAATA‐3′

Abbreviations: F, forward; GAPDH, glyceraldehyde phosphate dehydrogenase; HDAC3, histone deacetylase 3; miR‐495‐3p, microRNA‐495‐3p; R, reverse; TRAF5, tumour necrosis factor receptor‐associated factor 5.

### 2.13 Dual luciferase reporter gene assay

The target relation between miR‐495‐3p and TRAF5 was verified using dual luciferase reporter gene assay. TRAF5 3'UTR segment was synthetized and introduced into pMIR‐reporter (Ambion; Thermo Fisher Scientific Inc) to produce TRAF5 3'UTR wild‐type (WT) plasmid (TRAF5‐WT). Mutant sites of complementary sequence of the seed sequence were designed in TRAF5 3'UTR, and then, the target segment was inserted into pMIR‐reporter to produce TRAF5 3'UTR mutant type (MUT) plasmid (TRAF5‐MUT). The sequenced luciferase reporter plasmids wild type (WT) and mutant type (MUT) were, respectively, co‐transfected into A375 and SK‐MEL‐28 with mimics NC, miR‐495‐3p mimics. Transfected for 48 hours, the cells were collected and lysed. Luciferase detection kits (BioVision Incorporated.) and a fluorescence detector (Promega Corporation) were applied to determine the luciferase activity.

### 2.14 Chromatin immunoprecipitation (ChIP) assay

The experiment was in strict line with direction of EZ‐ChIP kits (Millipore Inc). A375 and SK‐MEL‐28 cells were incubated with 1% formaldehyde on a shaking table, and the crosslink was stopped by glycine after 10 minutes. Cells were centrifuged at 2000 rpm for 5 minutes and added with sodium dodecyl sulphate lysis buffer for ultrasonication, then centrifuged at 10 000 *g* and 4°C for 10 minutes. The product of ultrasonication (100 μL) was added with 900 μL ChIP dilution buffer, 20 μL 50 × pre‐initiation‐complex and 60 μL Protein A Agarose/SalmonSperm DNA, then mixed at 4°C for 1 hour and placed for 10 minutes. Cells were centrifuged at 700 rpm for 1 minutes, and 20 μL sample was taken as the input. A tube was added with 1 μL HDAC3 antibody and immunoglobulin G antibody, which were not added in the other tube. Tubes were incubated at 4°C overnight, and the samples were washed, eluted and decrosslinked. DNA sample was recycled and conducted with RT‐qPCR.

### 2.15 Subcutaneous tumorigenesis in nude mice

Forty‐two BALB/c male nude mice (Beijing Vital River Laboratory Animal Technology Co., Ltd.) aged 4‐6 w were randomly separated into two large groups: the A375 and SK‐MEL‐28 groups, which were, respectively, classified into seven small groups according to the cellular experiments. The nude mice were fed in specific pathogen‐free animal laboratory. A375 and SK‐MEL‐28 cells were detached, centrifuged, resuspended and made into cell suspension (1 × 10^7^ cells/mL). Each nude mouse was subcutaneously injected with 1 × 10^6^ cells according to the grouping. On the 7th day of the injection, the long and short diameters of the xenografts were measured. Mice were euthanized on the 35th day, and the xenografts were weighed. Tumour volume = long diameter × short diameter^2^/2.

### 2.16 Statistical analysis

All data analyses were conducted using SPSS 21.0 software (SPSS, IBM Corp). Relation between HDAC3/miR‐495‐3p expression and pathological characteristics of patients was analysed by chi‐square test, and the relation between HDAC3/miR‐495‐3p expression and prognosis of patients was analysed by Kaplan‐Meier survival analysis. The measurement data were expressed as mean ± standard deviation. The *t* test was performed for comparisons between two groups and one‐way analysis of variance (ANOVA) was used for comparisons among multiple groups, and Tukey's post hoc test was used for pairwise comparisons after one‐way ANOVA *P* value <.05 was indicative of statistically significant difference.

## 3 RESULTS

### 3.1 HDAC3 and TRAF5 are up‐regulated while miR‐495‐3p is down‐regulated in melanoma tissues and cells

**FIGURE 1:**
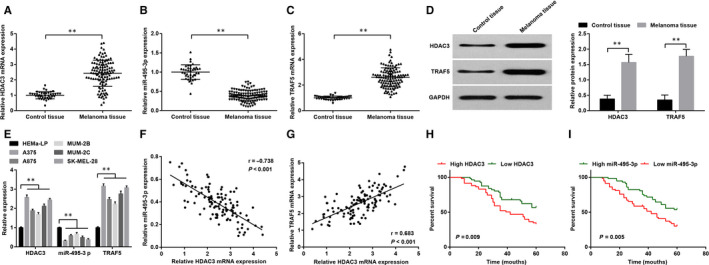
HDAC3 and TRAF5 are up‐regulated while miR‐495‐3p is down‐regulated in melanoma tissues and cells, and reduced miR‐495‐3p and overexpressed HDAC3 indicate a poor prognosis of melanoma patients. A, HDAC3 mRNA expression in melanoma tissues (n = 115) and pigmented nevus tissues (n = 40); B, miR‐495‐3p expression in melanoma tissues (n = 115) and pigmented nevus tissues (n = 40); C, TRAF5 mRNA expression in melanoma tissues (n = 115) and pigmented nevus tissues (n = 40); D, protein expression of HDAC3 and TRAF5 expression in melanoma tissues (n = 115) and pigmented nevus tissues (n = 40); E, mRNA expression of HDAC3, miR‐495‐3p and TRAF5 in cell lines; F, relation between expression of HDAC3 and miR‐495‐3p; G, relation between expression of HDAC3 and TRAF5; H, effect of HDAC3 on prognosis of melanoma patients; I, effect of miR‐495‐3p on prognosis of melanoma patients; *P < .05, **P < .01; the measurement data were expressed as mean ± standard deviation, the unpaired *t* test was performed for comparisons between two groups and the relation between HDAC3/miR‐495‐3p expression and prognosis of patients was analysed by Kaplan‐Meier survival analysis

Reverse transcription quantitative polymerase chain reaction and Western blot analysis were employed to measure the expression of HDAC3, miR‐495‐3p and TRAF5 in melanoma and pigmented nevus (control) tissues, and we found that (Figure 1A‐D) compared to the control tissues, HDAC3 and TRAF5 were up‐regulated while miR‐495‐3p was down‐regulated in melanoma tissues; HDAC3, miR‐495‐3p and TRAF5 expression in cells was assessed as well and we found that (Figure 1E) HDAC3 and TRAF5 were up‐regulated while miR‐495‐3p was down‐regulated in A375, A875, MUM‐2B, MUM‐2C and SK‐MEL‐28 cells, vs the HEMa‐LP cells (all *P < *.05). A375 and SK‐MEL‐28 cells were screened for subsequent experiments. Pearson correlation analysis was used to further verify the relationships between expression of HDAC3 and miR‐495‐3p/TRAF5 in the development of melanoma. The results indicated that (Figure 1F,G) the expression of HDAC3 and miR‐495‐3p was in a negative relation, while that of HDAC3 and TRAF5 was in a positive relation. Thus, we inferred that HDAC3 and miR‐495‐3p played essential roles in melanoma.

### 3.2 Reduced miR‐495‐3p and overexpressed HDAC3 indicate a poor prognosis of melanoma patients

We conducted the Kaplan‐Meier survival analysis to evaluate the predictive role of HDAC3/miR‐495‐3p expression in prognosis of patients with melanoma. Patients were separated into two groups according to the median of relative expression of HDAC3 and miR‐495‐3p: the low expression group and high expression group. The results of Kaplan‐Meier survival analysis showed that (Figure 1H,I) patients with high expression of HDAC3 or low expression of miR‐495‐3p had a poorer prognosis (both *P < *.05).

Relation between HDAC3/miR‐495‐3p expression and pathological features of melanoma patients was analysed. The results revealed that (Table 2) patients with III‐IV stage, Breslow's thickness ≥1.5 mm, LNM and distant metastasis had an increased ratio of high HDAC3 expression and low miR‐495‐3p expression (all *P < *.05), indicating that the expression of HDAC3 and miR‐495‐3p was related to TNM stage, Breslow's thickness, LNM and distant metastasis of patients (all *P < *.05), while was not correlated to age, gender, tumour classification and anabrosis (all *P >*.05).

**TABLE 2:** Relation between HDAC3/miR‐495‐3p expression and pathological features of melanoma patients

Pathological feature	n	HDAC3 expression		*P*	MiR‐495‐3p expression		*P*
		Low expression	High expression		Low expression	High expression	
		(n = 57)	(n = 58)		(n = 58)	(n = 57)	
Age (year)							
<60	48	26	22	.452	20	28	.132
≥60	67	31	36		38	29	
Gender							
Male	75	40	35	.329	34	41	.171
Female	40	17	23		24	16	
TNM stage							
I‐II	56	35	21	.015	18	38	<.001
III‐IV	59	22	37		40	19	
Tumour classification							
Acral type	41	22	19	.563	17	24	.176
Other	74	35	39		41	33	
Breslow's thickness							
<1.5 mm	43	28	15	.012	14	29	.004
≥1.5 mm	72	29	43		44	28	
Anabrosis							
Yes	63	27	36	.136	37	26	.062
No	52	30	22		21	31	
LNM							
Yes	64	24	40	.005	41	23	.001
No	51	33	18		17	34	
Distant metastasis							
Yes	42	14	28	.012	29	13	.004
No	73	43	30		29	44	

Note:

Data in the table were enumeration data and analysed by chi‐square test.

Abbreviations: HDAC3, histone deacetylase 3; HDAC3, miR‐495‐3p, microRNA‐495‐3p; LNM, lymph node metastasis; TNM, tumour node and metastasis.

### 3.3 HDAC3 mediates miR‐495‐3p to regulate TRAF5

**FIGURE 2:**
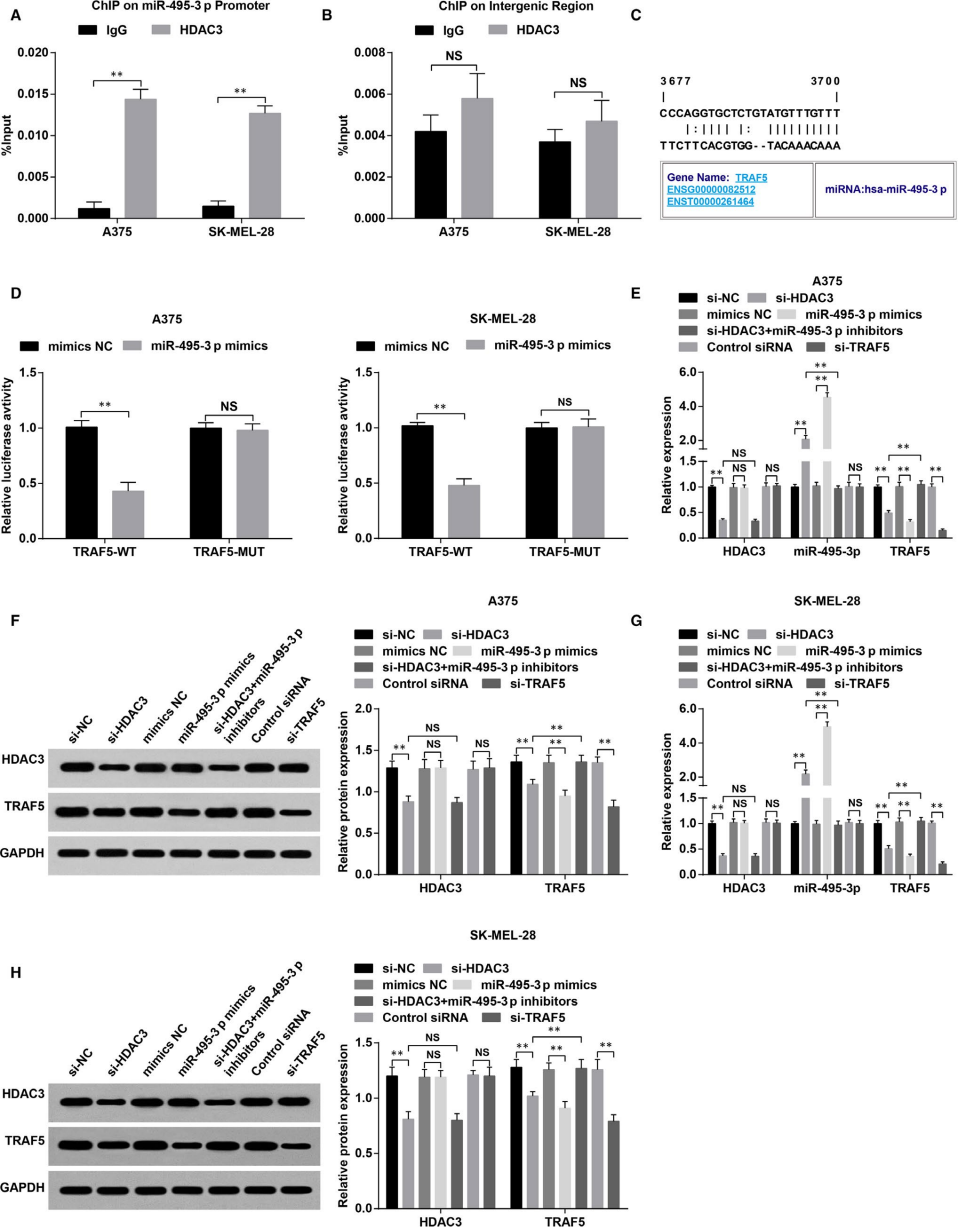
HDAC3 mediates miR‐495‐3p to regulate TRAF5 in melanoma cells. A, binding enrichment of HDAC3 and promoter of miR‐495‐3p in A375 and SK‐MEL‐28 cells was detected by ChIP assay; B, binding enrichment of HDAC3 and uncorrelated intergenic region in A375 and SK‐MEL‐28 cells was detected by ChIP assay; C, target relation between miR‐495‐3p and TRAF5 was predicted by a bioinformatic software; D, target relation between miR‐495‐3p and TRAF5 was confirmed by dual luciferase reporter gene assay; E, expression of HDAC3, miR‐495‐3p and TRAF5 in A375 cells of each group; F, protein expression of HDAC3 and TRAF5 in A375 cells of each group; G, expression of HDAC3, miR‐495‐3p and TRAF5 in SK‐MEL‐28 cells of each group; H, protein expression of HDAC3 and TRAF5 in SK‐MEL‐28 cells of each group; N = 3, *P < .05, **P < .01; the measurement data were expressed as mean ± standard deviation, the unpaired *t* test was performed for comparisons between two groups, one‐way ANOVA was used for comparisons among multiple groups and Tukey's post hoc test was used for pairwise comparisons after one‐way ANOVA

ChIP assay was applied to detect whether HDAC3 could bind to the promoter of miR‐495‐3p (Figure 2A,B). The results showed that HDAC3 was related to promoter of miR‐495‐3p, while was not related to uncorrelated intergenic region, suggesting that HDAC3 was able to directly regulate the expression of miR‐495‐3p.

It was predicted that by a bioinformatic software that there existed a target relation between miR‐495‐3p and TRAF5 (Figure 2C), and the outcomes of dual luciferase reporter gene assay indicated that (Figure 2D) miR‐495‐3p mimics suppressed the luciferase activity of WT TRAF5 vector, while did not affect that of MUT plasmid, indicating a targeting relationship between TRAF5 and miR‐495‐3p.

Results of RT‐qPCR and Western blot analysis reflected that (Figure 2E‐H) HDAC3 siRNA up‐regulated miR‐495‐3p expression whereas down‐regulated TRAF5 expression; miR‐495‐3p mimics inhibited TRAF5 expression; TRAF5 siRNA down‐regulated TRAF5 expression while didn't affect HDAC3/miR‐495‐3p expression; miR‐495‐3p inhibitors reversed the effect of si‐HDAC3 on TRAF5 expression. These results indicated that HDAC3 mediated miR‐495‐3p expression to regulate TRAF5 expression in melanoma cells.

### 3.4 Inhibited HDAC3 elevates miR‐495‐3p to repress growth of melanoma cells by down‐regulating TRAF5

**FIGURE 3:**
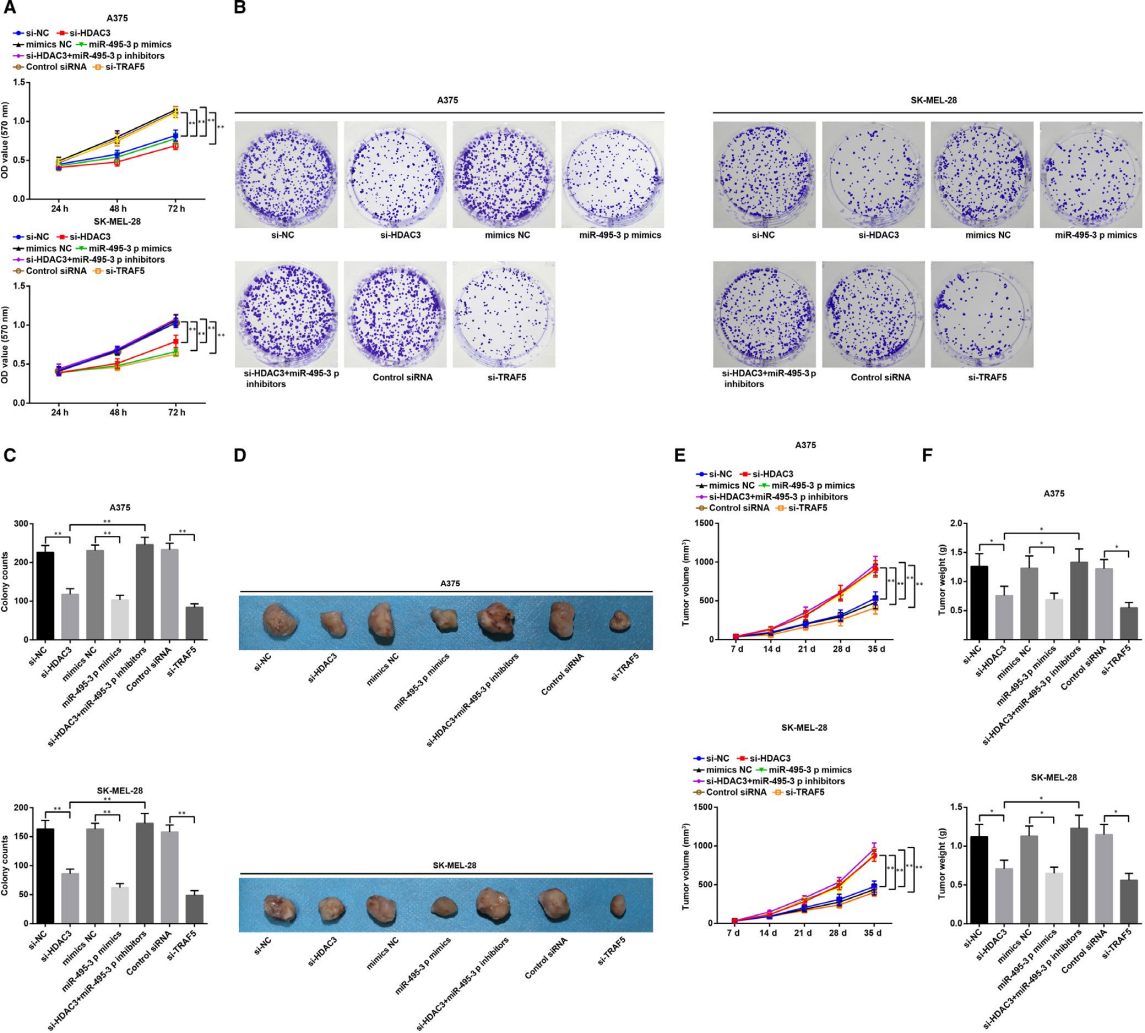
Inhibited HDAC3 elevates miR‐495‐3p to repress growth of melanoma cells by down‐regulating TRAF5. A, viability of A375 and SK‐MEL‐28 cells was determined by MTT assay; B, colony formation ability of A375 and SK‐MEL‐28 cells was determined by colony formation assay; C, number of colonies in each group; D, xenografts from nude mice that had been injected with A375 and SK‐MEL‐28 cells; E, volume of xenografts from nude mice that had been injected with A375 and SK‐MEL‐28 cells; F, weight of xenografts from nude mice that had been injected with A375 and SK‐MEL‐28 cells; N = 3, *P < .05, **P < .01; the measurement data were expressed as mean ± standard deviation, one‐way ANOVA was used for comparisons among multiple groups and Tukey's post hoc test was used for pairwise comparisons after one‐way ANOVA

We further discussed the role of HDAC3/miR‐495‐3p/TRAF5 in the biological functions of melanoma cells in vivo and in vitro using MTT assay, colony formation assay and subcutaneous tumorigenesis in nude mice. The results indicated that (Figure 3A‐F) HDAC3 siRNA, TRAF5 siRNA or miR‐495‐3p mimics suppressed the proliferation, colony formation ability and tumorigenesis of melanoma cells; the impacts of si‐HDAC3 were abolished by miR‐495‐3p inhibitors.

These findings mirrored that HDAC3 mediated miR‐495‐3p to regulate TRAF5 expression, thus affecting the growth of melanoma cells.

### 3.5 Down‐regulated HDAC3 elevates miR‐495‐3p to promote G0/G1 phase arrest and apoptosis of melanoma cells via repressing TRAF5

**FIGURE 4:**
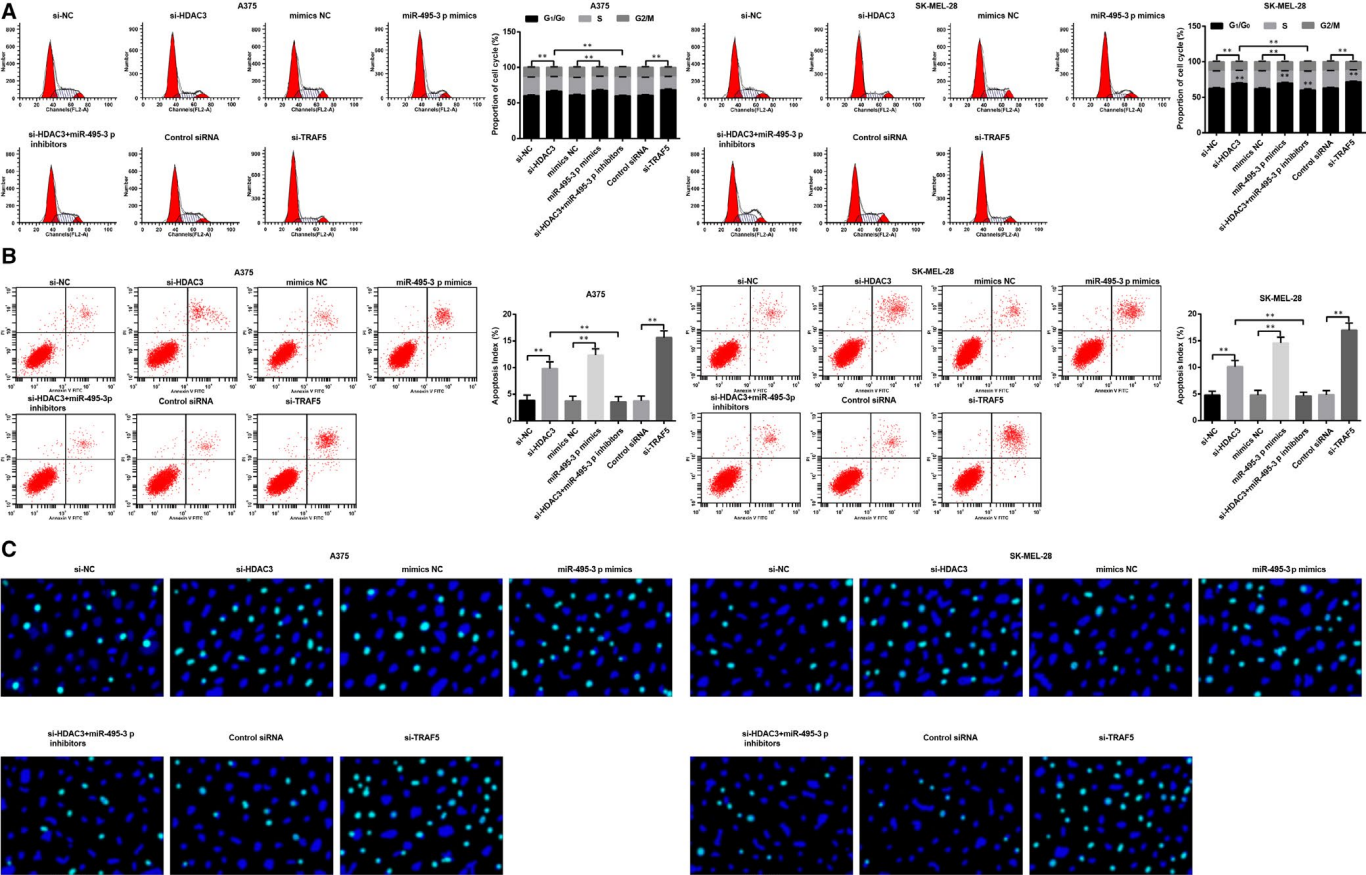
Down‐regulated HDAC3 elevates miR‐495‐3p to promote G0/G1 phase arrest and apoptosis of melanoma cells via repressing TRAF5. A, cell cycle distribution of A375 and SK‐MEL‐28 cells in each group was detected by flow cytometry; B, apoptosis of A375 and SK‐MEL‐28 cells in each group was detected by flow cytometry; C, morphology of A375 and SK‐MEL‐28 cells was observed through Hoechst 33 342 staining; N = 3, *P < .05, **P < .01; the measurement data were expressed as mean ± standard deviation, one‐way ANOVA was used for comparisons among multiple groups and Tukey's post hoc test was used for pairwise comparisons after one‐way ANOVA

We conducted flow cytometry to measure the cell cycle arrest and apoptosis rate of A375 and SK‐MEL‐28 cells. Outcomes unravelled that (Figure 4A‐D) si‐HDAC3, si‐TRAF5 or miR‐495‐3p mimics arrested cells at G0/G1 phase and promoted apoptosis; miR‐495‐3p inhibitors reversed the role of si‐HDAC3 on cell arrest distribution and apoptosis.

Hoechst 33342 entered cells through the cytomembrane and bound with DNA, which expressed as blue fluorescence under the excitation light source. A larger amount of Hoechst 33342 entered apoptotic cells due to the incomplete cytomembranes, and there appeared brighter blue fluorescence. It was observed in Hoechst 33342 staining that (Figure 4E) cells in the si‐HDAC3, miR‐495‐3p mimics and si‐TRAF5 groups performed apparent apoptotic characteristics compared to their NC groups: the nuclei were shrunk and lysed much completely, there were dense massive lumpy or granular fluorescence in nuclei, increased apoptotic cells and decreased normal cells. Contrasted to si‐HDAC3 group, there were evenly distributed blue fluorescence and markedly decreased apoptotic cells in the si‐HDAC3 + miR‐495‐3p inhibitors, si‐NC, mimics NC and Control siRNA groups. These results were in accordance with that of flow cytometry.

### 3.6 Declined HDAC3 increases miR‐495‐3p to inhibit migration, invasion and epithelial‐mesenchymal transition (EMT) of melanoma cells through reducing TRAF5

**FIGURE 5:**
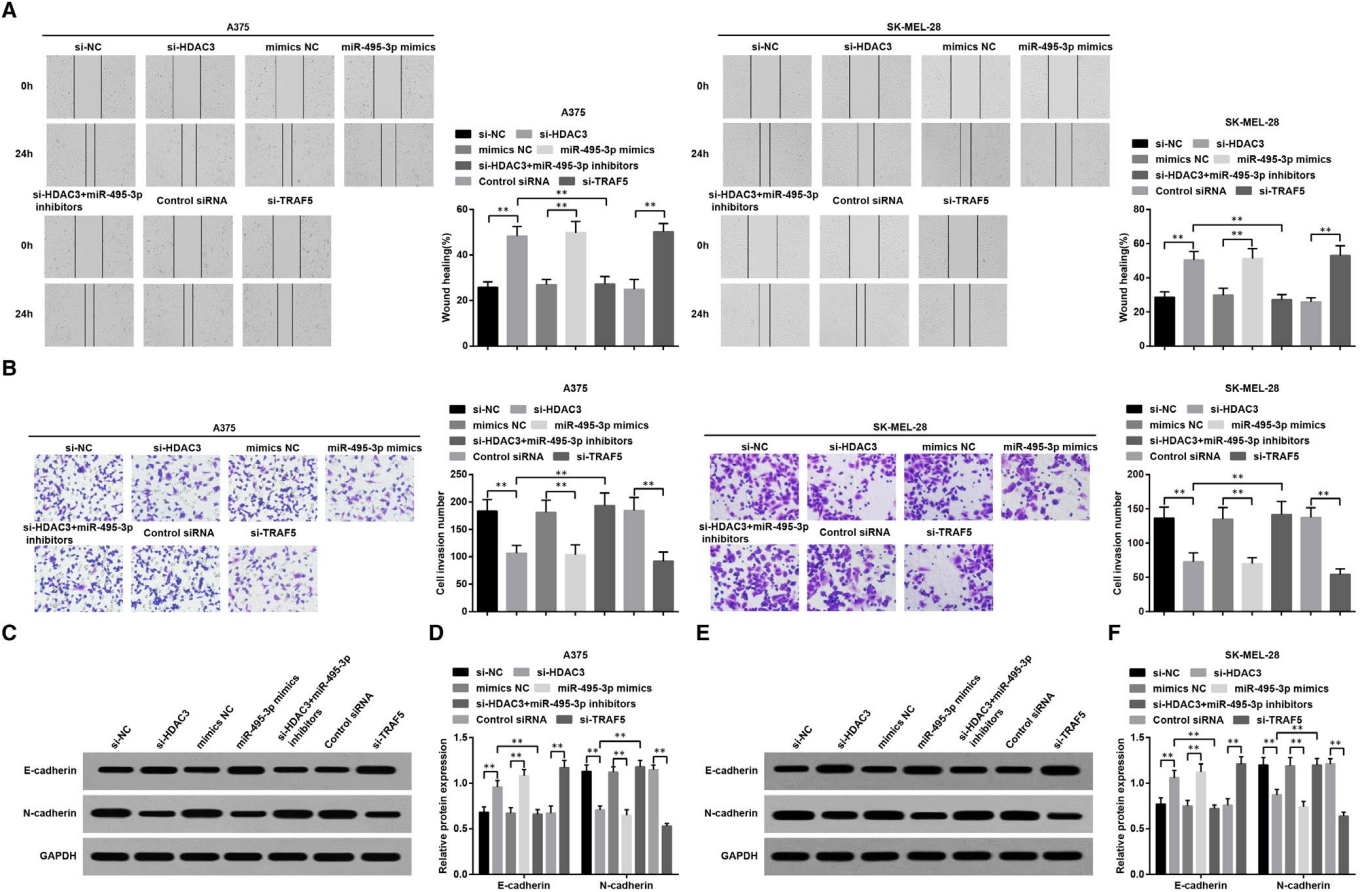
Declined HDAC3 increases miR‐495‐3p to inhibit migration, invasion and EMT of melanoma cells through reducing TRAF5. A, migration ability of A375 and SK‐MEL‐28 cells in each group was detected by scratch test; B, invasion ability of A375 and SK‐MEL‐28 cells in each group was detected by Transwell assay; C, protein bands of E‐cadherin and N‐cadherin in A375 cells of each group; D, protein expression of E‐cadherin and N‐cadherin in A375 cells of each group; E, protein bands of E‐cadherin and N‐cadherin in SK‐MEL‐28 cells of each group; F, protein expression of E‐cadherin and N‐cadherin in SK‐MEL‐28 cells of each group; N = 3, *P < .05, **P < .01; the measurement data were expressed as mean ± standard deviation, one‐way ANOVA was used for comparisons among multiple groups and Tukey's post hoc test was used for pairwise comparisons after one‐way ANOVA

Scratch test and Transwell assay were utilized to determine the migration and invasion abilities of A375 and SK‐MEL‐28 cells, and we observed that (Figure 5A‐D) in contrast to the relative NC groups, the migration and invasion abilities of A375 and SK‐MEL‐28 cells were both suppressed in the si‐HDAC3, miR‐495‐3p mimics and si‐TRAF5 groups; the inhibitive impact of reduced HDAC3 could be reversed by inhibition of miR‐495‐3p. The protein expression of E‐cadherin and N‐cadherin was detected using Western blot analysis, and it was found that (Figure 5E‐H) si‐HDAC3, miR‐495‐3p mimics or si‐TRAF5 up‐regulated E‐cadherin expression while down‐regulated N‐cadherin expression, and the role of si‐HDAC3 was abrogated by miR‐495‐3p inhibitors. These results showed that HDAC3 mediated miR‐495‐3p to regulate TRAF5 expression and then promoted the migration, invasion and EMT progression of melanoma cells

## 4 DISCUSSION

Melanoma is a class of tumour cells that originates from melanocytes and initially develops in skin. Recently, the prevalence of melanoma has enhanced in the world.^17^ HDAC3 is a multimolecular complex that includes the nuclear receptor co‐repressor 1 and silencing mediator of retinoid and thyroid hormone receptor protein subunits, which are required for the physiological action of many nuclear hormone receptors.^18^ This study was designed to investigate effect of the HDAC3/miR‐495‐3p/TRAF5 axis in the development of melanoma, and we found that the inhibition of HDAC5 has the ability to elevate miR‐495‐3p to restrain the progression of melanoma by reducing TRAF5.

The expression levels of HDAC3, miR‐495‐3p and TRAF5 in melanoma cells and tissues were determined, and the results implied that HDAC3 and TRAF5 were up‐regulated while miR‐495‐3p was down‐regulated in melanoma cells and tissues, respectively, contrasted to normal melanocytes and pigmented nevus tissues. The abnormal expression levels have also been clarified in other studies. For example, Xia et al^13^ have suggested that miR‐495‐3p was markedly decreased in melanoma cells, and a former study has revealed that HDAC3 was highly expressed in melanoma samples, especially in tumour‐invaded regions, in relation to non‐melanoma samples.^8^ Additionally, Li et al^16^ demonstrated that the expression TRAF5 was higher in melanoma tissues than in normal tissues. We also provided evidence that HDAC3 was able to negatively regulate miR‐495‐3p and TRAF5 was targeted by miR‐495‐3p, while both of the relationships were seldom studied.

The relation between HDAC3/miR‐495‐3p expression and pathological characteristics of melanoma patients was analysed, and it was found that the expression of HDAC3 and miR‐495‐3p was related to TNM stage, Breslow's thickness, LNM and distant metastasis of patients. Similarly, Mao et al^19^ have affirmed that the low expression of miR‐495 was related to LNM, invasion and TNM stage of patients with oesophageal squamous cell carcinoma, and it has been clarified that high expression of HDAC3 was positively corresponded with advanced TNM stage of patients with breast cancer.^20^ Furthermore, results of cellular experiments in our study indicated that inhibited HDAC3 was able to elevate miR‐495‐3p to decelerate proliferation, migration and invasion of melanoma cells, and also induced G0/G1 phase arrest and accelerate melanoma cell apoptosis. In accordance with the results, Shan et al^8^ have pointed out that the reduction of HDAC3 had the capacity of inhibiting proliferation of melanoma cells and arresting melanoma cells at G0/G1 phase, and it has been recently reported that HDAC3 silencing resulted in promoted killing of melanoma cells.^9^ Moreover, a recent publication has indicated that overexpressed miR‐495‐3p decelerated proliferation, invasion and migration of melanoma cells,^13^ and Chen et al^14^ have also unearthed that miR‐495 suppressed proliferation, invasion and migration, and promoted apoptosis of melanoma cells. As for the effect of TRAF5 on biological processes of melanoma cells, Li et al^16^ have identified that the repression of TRAF5 was accompanied by inhibited proliferation and enhanced apoptosis of melanoma cells. Except for in vitro experiments, we also conducted in vivo experiment to probe into the roles of HDAC3, miR‐495‐3p and TRAF5 in melanoma cell growth. The outcomes implied that down‐regulation of HDAC3 elevated miR‐495‐3p to suppress tumour growth in vivo through declining TRAF5. Consistently, Lu et al^21^ have figured out that reduced HDAC3 inhibited xenograft tumour growth in liver cancer, and Cui et al^22^ have suggested that miR‐495‐3p contributed to restraining tumour growth in gastric cancer. In addition, we demonstrated that inhibited HDAC3 enhanced miR‐495‐3p to restrict EMT in melanoma by silencing TRAF5. In line with this finding, it has been unveiled that knockdown of HDAC3 constrained EMT of cutaneous squamous cell carcinoma,^23^ and a research has clarified that miR‐495‐3p was a negative regulator of EMT in fibrosis formation.^24^

In summary, we demonstrated that reduced HDAC3 was able to up‐regulate miR‐495‐3p to suppress malignant behaviours of melanoma cells, thereby decelerating the development of melanoma with the involvement of TRAF5. FK228 (depsipeptide) is a HDAC inhibitor with pleiotropic antitumor activities ^25^ which can induce apoptosis in melanoma cells.^26^ Thus, FK228 can be used to down‐regulate HDAC3 to repress the development of melanoma, which could be explored in our future research. This study may contribute to exploration on therapeutic strategies for melanoma. Nevertheless, the detailed molecular mechanisms remain to be explored.

## CONFLICT OF INTEREST

The authors declare that they have no conflicts of interest.

## AUTHOR CONTRIBUTION

**Yanbo Ma:** Writing‐review & editing (equal). **Jincheng Duan:** Investigation (equal); Methodology (equal). **Xiuyan Hao:** Conceptualization (equal); Data curation (equal).

## Data Availability

The data that support the findings of this study are available from the corresponding author upon reasonable request.
